# Development of an Immunoassay for the Detection of Copper Residues in Pork Tissues

**DOI:** 10.3390/bios11070235

**Published:** 2021-07-13

**Authors:** Junqiu Zhang, Liwei Xu, Hongtao Jiang, Chuanlai Xu, Wenjing Liu, Ting Wu, Hua Kuang

**Affiliations:** 1 International Joint Research Laboratory for Biointerface and Biodetection, State Key Lab of Food Science and Technology, School of Food Science and Technology, Jiangnan University, Wuxi 214122, China; zjq1413@126.com (J.Z.); xuliwei0529@163.com (L.X.); jianghongtao@stu.jiangnan.edu.cn (H.J.); xcl@jiangnan.edu.cn (C.X.); 2 Shanxi Academy of Analytical Sciences, No. 17 Beiyuan Street, Taiyuan 030012, China; liuwj060@163.com (W.L.); cau6281@126.com (T.W.)

**Keywords:** Cu, detection, ELISA, pig tissues

## Abstract

The presence of high concentrations of copper (Cu) residues in pork is highly concerning and therefore, this study was designed to develop a high-throughput immunoassay for the detection of such residues in edible pork tissues. The Cu content in the pork samples after digestion with HNO_3_ and H_2_O_2_ was measured using a monoclonal antibody (mAb) against a Cu (II)–ethylenediaminetetraacetic acid (EDTA) complex. The resulting solution was neutralized using NaOH at pH 7 and the free metal ions in the solution were chelated with EDTA for the immunoassay detection. An indirect competitive enzyme-linked immunosorbent assay (ic-ELISA) method was developed for Cu ion analysis. The half maximal inhibitory concentration of the mAb against Cu (II)–EDTA was 5.36 ng/mL, the linear detection range varied between 1.30 and 27.0 ng/mL, the limit of detection (LOD) was 0.43 μg/kg, and the limit of quantification (LOQ) was 1.42 μg/kg. The performances of the immunoassay were evaluated using fortified pig serum, liver, and pork samples and had a recovery rate of 94.53–102.24%. Importantly, the proposed immunoassay was compared with inductively coupled plasma mass spectroscopy (ICP-MS) to measure its performance. The detection correlation coefficients of the three types of samples (serum, pork, and liver) were 0.967, 0.976, and 0.983, respectively. Thirty pork samples and six pig liver samples were collected from local markets and Cu was detected with the proposed ic-ELISA. The Cu content was found to be 37.31~85.36 μg/kg in pork samples and 1.04–1.9 mg/kg in liver samples. Furthermore, we detected the Cu content in pigs with feed supplemented with tribasic copper chloride (TBCC) and copper sulfate (CS) (60, 110, and 210 mg/kg in feed). There was no significant difference in Cu accumulation in pork tissues between the TBCC and CS groups, while a remarkable Cu accumulation was found for the CS group in liver at 210 mg/kg, representing more than a two-fold higher level than seen in the TBCC group. Therefore, the proposed immunoassay was found to be robust and sensitive for the detection of Cu, providing a cost effective and practical tool for its detection in food and other complicated samples.

## 1. Introduction

Copper (Cu) is an essential trace element for all animals and it plays a vital role in the growth of livestock [1,2]. As an efficient and inexpensive growth promoter, the addition of high Cu concentrations has been widely used in animal feeds, including pig production, to increase growth. Tribasic Cu chloride (TBCC) and Cu sulfate (CS) have been widely used as Cu sources [3,4]. However, high Cu in feed and in tissue residues may lead to metabolic disturbances in pigs [5,6]. As early as 1981, Buntain showed that the use of high Cu as a growth promoter increased the incidence of gastric ulcers in intensive pig farming [7]. Excessive concentrations of Cu ions in vivo cause a change in the redox status of cells, leading to toxicity [8], as well as damaging DNA, proteins, and lipids [9]. Furthermore, high concentrations of Cu ions react nonspecifically with the side chains of amino acid residues, and can lead to the misfolding of proteins [10]. Uncombined Cu ions also compete with other substances for the active centers of enzymes, thus interfering with their normal function [11]. Some researchers have found that the accumulation of Cu in the human body may lead to atherosclerosis and rapid cell aging and death, posing a huge threat to public health [12,13,14]. In addition, Cu excretion from livestock has resulted in ecological concerns [15,16] and this increase in Cu in the environment can lead to soil degradation and water pollution, and can affect the growth rate of plants [17,18,19,20]. Therefore, some countries, including China, have set maximum limits to Cu supplements in fodder. According to the Announcement No. 2665 of the Ministry of Agriculture of China, the dietary Cu requirement for adult pigs is 3–6 mg/kg. Moreover, in the regional standard of safety sanitation and high-quality pork in Shanghai, China, the limit of copper is 10 mg/kg. In addition, the provisional maximum tolerable daily intake (PMTDI) for Cu determined by FAO/WHO is 0.5 mg (kg bw)^−1^ day^−1^. The average daily intake is estimated at 2–3 mg for adult persons [21]. Thus, our method can be used for actual sample detection. If the sample contains excess copper, it should be tested after proper dilution. This method can ensure the security of excessive copper in meat consumption.

Many types of qualitative and quantitative methods have been used to monitor Cu levels in food and the environment (Table 1 [22,23,24,25,26,27,28,29,30,31,32,33,34,35,36,37,38,39,40]), such as graphite furnace atomic absorption spectrometry to detect Cu levels in cattle and fish tissues [32]. Furthermore, the measurement of Cu levels in wild pig liver by flame atomic absorption spectrometry (FAAS) found that this organ is a rich source of Cu [33]. FAAS has also been used to detect Cu in pork viscera and other tissues from hybrid pigs [34] and inductively coupled plasma mass spectrometry (ICP-MS) has been used to determine the Cu content of pork and their internal organs [35]. These methods generally have the advantages of high sensitivity, but also have the disadvantages of relying on large instruments and high measurement costs.

Immunoassays such as enzyme-linked immunosorbent assays (ELISAs), however, are sensitive, fast, and cost-effective methods, which have been successfully applied to research in many fields such as the food industry, the environment, and clinical research [41,42,43,44,45]. However, it is a great challenge to develop an immunoassay for the detection of metal ions because there are no spatial antigenic epitopes with metal ions. In recent years, researchers have found a way to create specific antigenic determinants for targeting metal ions. They used chelating agents to capture metal ions and the resulting complex gave unique antigenic conformations, which could be used to screen specific antibodies, such as monoclonal antibodies (mAbs), directed to Pb (II) [46]. An immunochromatographic strip assay has also been developed with a mAb against mercury in water [47] and the development of a mAb against cadmium (Cd), as well as a highly sensitive Cu (II) enzyme-linked immunoassay, which successfully detected Cu in drinking water [48,49]. Furthermore, bifunctional chelating agents have also been used to prepare mAbs against Cu ions, which were used to establish an ELISA [50,51].

**Figure 1:**
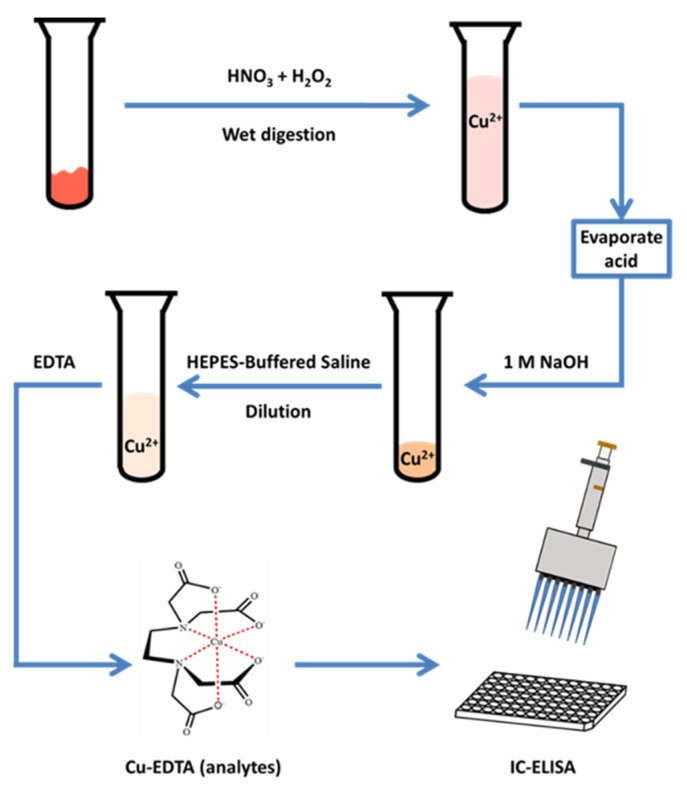
Immunoassay procedure for copper residues in animal tissues.

Although there exist a few examples of immunoassays for the detection of Cu, they focus on simple substrates such as water. Few immunoassays have been developed for meat, liver, and other complicated food substrates. Therefore, in this study, we developed an immunoassay for the detection of Cu in animal samples. The Cu ions in animal tissue samples were extracted and chelated with ethylenediaminetetraacetic acid (EDTA), and the resulting complex was detected with an ELISA method (Figure 1). Then, local meat and swine samples with inorganic Cu supplements were tested to determine the reliability and portability of the newly developed immunoassay. 

## 2. Materials and Methods

### 2.1. Instruments and Chemicals

Inductively coupled plasma mass spectrometry (NexION 350D, PerkinElmer, Shelton, CT, USA) and a graphite-digestion device (SH230N, Hanon, Jinan, China) were employed for sample preprocessing and analysis. The spectral absorbance of microwell plates was measured with a microplate reader (Eon, BioTek, Winooski, VT, USA). All chemicals were ultrapure grade and Cu (II) (1000 μg/mL in 1% HNO_3_) was purchased from the National Institute of Metrology (Beijing, China). Isothiocyanobenzyl-EDTA (ITCBE) was purchased from Dojindo Laboratories (Shanghai, China). Keyhole limpet hemocyanin (KLH), bovine serum albumin (BSA), and Freund’s complete and incomplete adjuvants were purchased from Sigma-Aldrich (St. Louis, MO, USA). Goat anti-mouse IgG (Fc specific) conjugated to horseradish peroxidase (HRP) was from Jackson ImmunoResearch (West Grove, PA, USA). A monoclonal antibody exhibiting specificity for the Cu (II)–EDTA complex was from our laboratory [49]. All plasticware was soaked overnight in 3 M HCl and glassware was acid washed and rinsed thoroughly with purified water before use. Water was purified by a Millipore purification system (Bedford, MA, USA).

### 2.2. Buffers and Solutions

The buffers and solutions used were: (1) phosphate-buffered saline (PBS): 137 mM NaCl, 10 mM phosphate, and 3 mM KCl, pH 7.4; (2) carbonate-buffered solution (CBS): coating solution, 100 mmol/L Na_2_CO_3_–NaHCO_3_, pH 9.6; (3) blocking buffer: 0.1% (*w/v*, g/L) gelatin in PBS; (4) 2-[4-(2-hydroxyethyl)-1-piperazine] ethanesulfonic acid (HEPES, CAS:7365-45-9)-buffered saline (HBS): 10 mM HEPES, 137 mM NaCl, and 3 mM KCl; and (5) washing solution (PBST): 0.01% (*v/v*) Tween 20 in PBS.

### 2.3. Sample Pretreatment

A total of 30 pork and 6 liver samples of pig were collected from local markets, and the other pig samples (include 36 of serum, pork, and liver, respectively) were kindly donated by Professor He Pingli’s laboratory at the Chinese Agricultural University, from which weanling pigs were fortified with two mineral source supplements (TBCC and CS) through an entire 38 day experiment. Three supplemented Cu concentrations, 60, 110, and 210 mg/kg, in the diet were given to the TBCC and CS pig groups.

Pig samples (serum, pork, and liver) were prepared for the analysis of Cu concentrations, which included wet tissue weights: 0.20 g of liver, pork, and serum samples were placed into digestion tubes. Then, 3 mL of nitric acid was added at 120 °C, and the samples were allowed to digest for 40 min, and then 1.2 mL of hydrogen peroxide was added for a further 40 min. After the digestion, samples were transferred to beakers and the acid was removed using an electric hot plate. The pH was then adjusted to 7.2 with 1 mol/L NaOH, and was used as a blank control.

The ICP-MS conditions for Cu detection are shown in SI.

### 2.4. Ic-ELISA Method for the Determination of Cu Ions

The ic-ELISA method was applied as previously published [49]. With a pH of 7.2, optimal IC_50_ sensitivity and maximum absorbance signals were obtained. Furthermore, 1 mM EDTA and HEPES-KCl assay buffer containing 137 mM NaCl, 3 mM KCl, and 10 mM HEPES were used in the assay. In this study, parameters such as incubation temperature, incubation time, and plate-washing times were optimized and the specific operational procedures were as follows.

Microtiter plates (96-well) were coated with Cu (II)–ITCBE–BSA at 1.5 mg/mL in 100 μL CBS (pH 9.0) at 37 °C for 2 h. After three washes with PBS (pH 7.4, 137 mM NaCl, 3 mM KCl, and 10 mM phosphate) containing 0.05% Tween 20 (PBST), the plates were blocked with blocking buffer for 2 h at 37 °C. The plates were then washed again and air-dried at 37 °C for 15 min. Then, checkerboard assays were employed to determine the optimal concentrations of coated Cu (II)–ITCBE–BSA and Cu (II) mAb for use in competitive assays. Cu (II)–EDTA mixture was prepared (0, 0.2, 0.6, 2, 7, 25, 80, and 240 ng/mL) in HBS (50 μL/well) and mixed with diluted purified antibody (50 μL/well) in the assay plate pre-coated with Cu (II)–ITCBE–BSA. The mixture was shaken for 20 s and incubated at 37 °C for 30 min. The plate was then washed, and 100 μL of the goat anti-mouse IgG conjugated with horseradish peroxidase (GAM-HRP) diluted 1:3000 with PBST was added in each well and incubated at 37 °C for 30 min. The plate was again washed to remove excess GAM-HRP, and 100 μL of TMB substrate solution was added to each well. After a 15 min incubation at 37 °C, the reaction was stopped by the addition of 50 μL of 2 M H_2_SO_4_ and the absorbance measured at 450 nm. In the ic-ELISA format, analytes that bind the antibody cause a decrease in the amount of antibody captured by the immobilized Cu (II)–ITCBE–BSA, resulting in reduced absorbance. Standard curves were obtained by plotting absorbance versus concentration (logarithm) of the standard Cu (II) analyte concentration and fitting the data to a four-parameter logistic equation. The spiked samples including pork, liver, and serum were employed for recovery test at three levels (*n* = 6). All samples were detected by ic-ELISA for background content of Cu, then the suitable volume of Cu (II) solution for fortification was added as follows: 0.5, 1.0, and 2.0 mg/kg in pork, 0.75, 1.5, and 3.0 mg/kg in serum, and 5, 10, and 20 mg/kg in liver samples.

In detection of real samples, the Cu standard solutions were replaced by diluted pig samples without fortification, while the rest of the experiment was performed as described above.

## 3. Results and Discussion

### 3.1. Optimization of ic-ELISA Conditions and Correlation Analysis with ICP-MS

**Figure 2:**
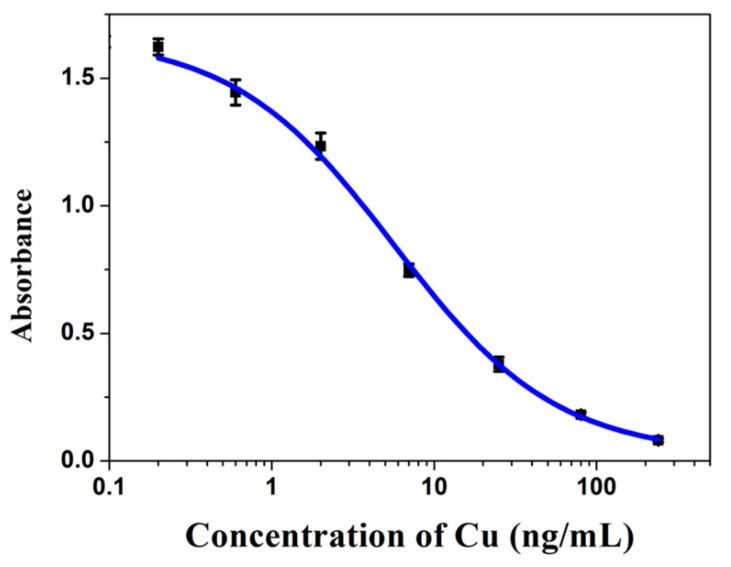
Standard inhibition curve for Cu ions by ic-ELISA method (*n* = 3).

Competitive inhibition curves of the Cu (II) analyte were obtained under optimal conditions as shown (Figure 2). The IC_50_ for the detection of Cu was determined to be 5.36 ± 0.51 ng/mL according to the standard calibration curve (Figure 2), the linear range was from 1.30 ± 0.09 to 27.0 ± 1.8 ng/mL, the limit of detection (LOD) was 0.43 μg/kg, and the limit of quantification (LOQ) was 1.42 μg/kg.

**Figure 3:**
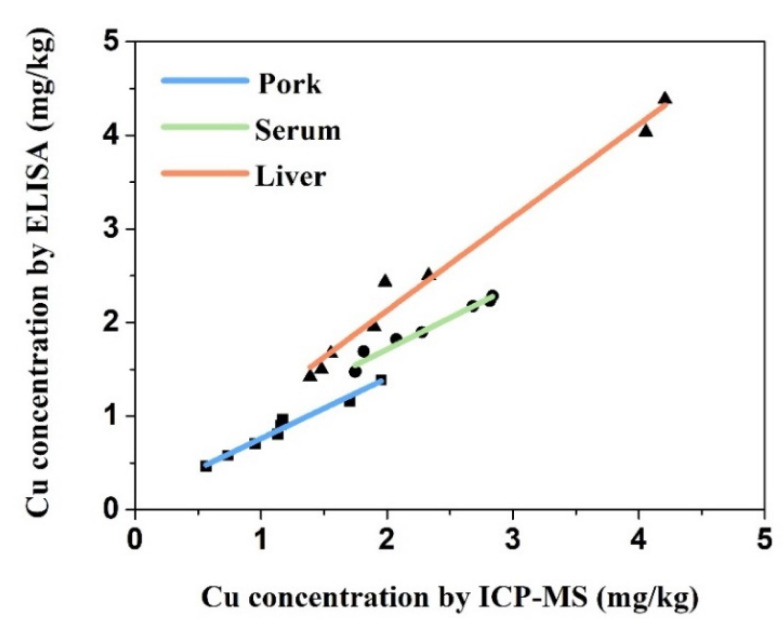
Comparison between ic-ELISA and ICP-MS for Cu residues in swine liver, serum, and pork samples (▴ Liver: y = x + 0.141, R^2^ = 0.983; ● serum: y = 0.673x + 0.369, R^2^ = 0.967; ■ pork: y = 0.645x + 0.118, R^2^ = 0.976).

To evaluate the practicality and accuracy of our ic-ELISA method, pig samples spiked with three concentrations of Cu (II) were analyzed, which followed the same process shown in Figure 1. As shown in Table 2, the Cu recovery from pork samples ranged from 94.53 to 102.24%, and the coefficients of variance (CV) were from 2.63 to 9.53%. The results show that the test had stable recovery and reproducibility. Furthermore, the performance of the immunoassay was evaluated by comparing it with ICP-MS detection. Three types of samples (pork, serum, and liver) were treated and analyzed. As a result, we developed a linear regression model for correlation analysis of the ELISA and ICP-MS methods (Figure 3). The correlation coefficient was 0.967 for pork, 0.976 for serum, and 0.983 for liver, indicating high reliability of this ic-ELISA method.

So far, many methods of detecting copper in liver, serum, and pork have been established. Some previously reported studies showed the LOD of Cu was 0.022 (ICP-MS) [52] and 0.5 mg/kg (ASS) [24] in liver and meat. Compared with other conventional techniques, this immunoassay has the advantage of low LOD. Moreover, the microplate of the ic-ELISA method makes the assay a high-throughput analysis, which compresses time for the detection of a large number of samples at the same time.

### 3.2. The Investigation of Cu Content in Retail Swine Samples

We randomly collected 30 pork and 6 liver samples from local markets and all were treated and analyzed. The Cu levels were found to be 37.60~85.36 μg/kg in pork, and 1.04~1.90 mg/kg in liver samples, and the results show much higher bioaccumulation of Cu in liver than in pork tissue. (Table 3). Cu is essential to several enzymes responsible for metabolic processes in vivo [52,53]. In addition, some research suggests that an overdose of Cu can lead to its accumulation in the liver and potentially result in health risks. Cu accumulation in liver is possibly related to the distribution of Cu-containing enzymes in animals or metabolic pathways associated with Cu [1,2].

### 3.3. Distributions of Cu in Pigs under Different Treatments

**Figure 4:**
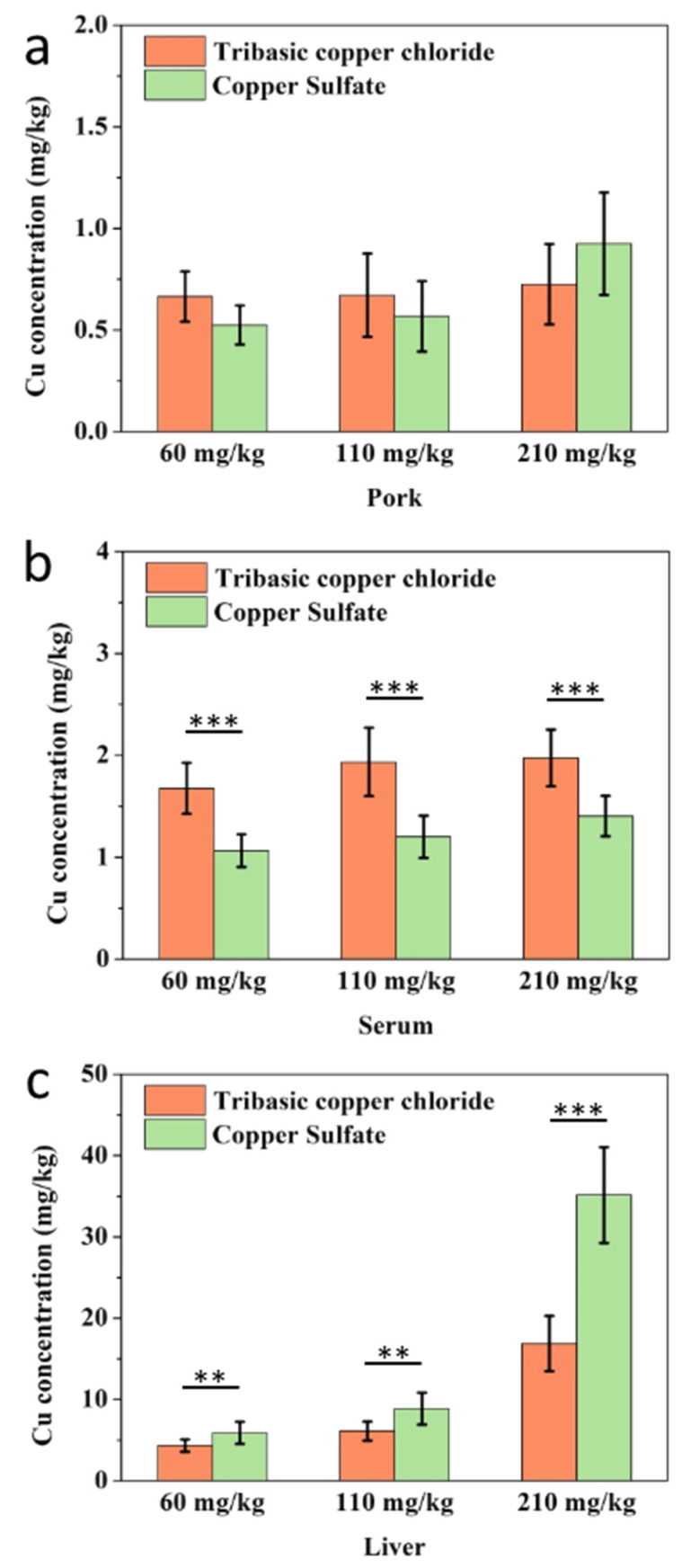
Cu concentration in pork, serum, and liver sample by ic-ELISA for (**a**) copper concentration in pork; (**b**) copper concentration in serum; (**c**) copper concentration in liver. (*: *p* < 0.05, **: *p* < 0.01, ***: *p* < 0.001). *p*-values represent the difference among three groups as determined by Student’s *t*-test.

To further investigate the application of our developed immunoassay, we analyzed the Cu content in swine samples (donated sample) after supplementation with TBCC or CS from the diet. As shown in Figure 4a, no significant difference in total Cu content in pork between the two mineral types of Cu was seen. The Cu residues in pork ranged from 0.5 to 1 mg/kg, the Cu content in serum samples was found to be up to 2.38 ± 0.19 mg/kg (Table S1), and the Cu levels from the TBCC groups were approximately 1.4 times higher than the CS groups. (*p* < 0.001). However, there were no obvious dosage effects among the 60, 110, and 210 mg/kg fortified levels in the two Cu-type treatment groups. This may be due to Cu being absorbed into proteins in the blood and released into other tissues after reaching the bearing capacity (Figure 4b). It should be noted that a remarkable Cu accumulation was found in swine liver, which was dose dependent (Figure 4c). Furthermore, compared to the TBCC treatment group, the CS diet group led to higher residues in liver tissue. As a result, the Cu level of the CS group at 210 mg/kg group (35.06 ± 5.27 mg/kg) was more than six-fold higher than the 60 mg/kg group and four-fold greater than that of the 110 mg/kg group. which was also more than two times lower than the TBCC group at 210 mg/kg (* *p* < 0.001).

According to previously published data, Cu in fodder is absorbed into the blood through the intestinal tract, and mainly conjugates with plasma proteins. Cu intake by livestock is distributed in erythrocytes and plasma, and mainly manifests in the form of erythrocyte cupratin and ceruloplasmin [54,55,56,57]. When Cu overdose occurs, it is transported to the liver for storage, which results in the bioaccumulation of Cu in vivo [58,59,60]. The Cu concentrations in tissue and liquid samples indicated that the liver was a target organ and was sensitive to changes in dietary Cu content (Figure 4). Therefore, liver Cu levels could be used as a sensitive index of excessive Cu in pig products. The data show that the two inorganic Cu-sourced feed supplements underwent different metabolic pathways in animals (Supplementary Material Table S1). Therefore, an assessment of dietary Cu supplements in animals should be carried out to more fully understand its bioavailability [61,62]. 

## 4. Conclusions

In this study, we developed a rapid and high-throughput immunoassay for the detection of Cu in complicated pork, liver, and serum matrices. The result of our ELISA analysis was found to be highly consistent with other methods, such as ICP-MS. The LOD value (µg per kg sample) was far lower than the Cu residue level of pig samples (mg per kg sample). Copper analysis in local pork tissues indicated that its residues in pork was low, but its accumulation in the liver was much more serious. In pigs, we found different accumulation features for Cu depending upon its tribasic TBCC and sulfated CS forms. The latter showed a significant retention in swine liver. Therefore, it is necessary to carry out a systematic study of Cu metabolism in farmed livestock, and its environmental evaluation.

## Figures and Tables

**Table 1:** Detection method of Cu content in different samples.

Detect Method	Sample Type	LOD (μg/kg, mg/kg *)	LOQ (μg/kg, mg/kg *)	Reference
ICP-MS	Olive	0.03	0.07	[22]
	Tomato paste	0.16	0.53	[23]
	Leaves	9		[24]
	Fruit wines	7.13 × 10^−3^	23.8 × 10^−3^	[25]
	Bovine liver	0.022 *	0.066 *	[26]
ICP-OES	Sugarcane juice	0.04 *	0.13 *	[27]
	Oil	2.1 *	6.8 *	[28]
	Tea	0.06 *	0.2 *	[29]
	Human serum	0.08	0.28	[30]
AAS	Grape marc distillate	0.097 *	0.322 *	[31]
	Bovine liver	1.8 *	6.0 *	[32]
	Salmon	2.0 *	6.6 *	
	Rolled oats	1.4 *	4.6 *	
	Wild boar liver		2.6 *	[33]
	Pig semimembranosus muscle	0.5 *	0.75 *	[34]
	Pig liver	0.5 *	0.75 *	
	Pig longissimus thoracis et lumborum	0.022 *	0.066 *	[35]
	Pig liver	0.022 *	0.066 *	
	Pig kidney	0.022 *	0.066 *	
Biosensor	Cu (II) stock solutions	0.71		[36]
	Aqueous solutions.	64		[37]
	Sweat; serum	3	10	[38]
	Mine	0.06		[39]
	Mice liver	0.9*		[40]
	Mice urinary	0.9*		

LOD: Limit of detection. LOQ: Limit of quantification. * in Table 1 refer the unit of data to mg/kg.

**Table 2:** Recovery test of Cu (II) concentration in pig samples (*n* = 6).

Sample	Original Concentrations (mg/kg)	Fortified Cu (II) Level (mg/kg)	Detection by ELISA (Mean ± SD) (mg/kg)	Recovery (%)	CV (%)
Pork	1.07	0.50	1.56 ± 0.14	99.24	8.35
	1.07	1.00	2.10 ± 0.16	101.23	6.84
	1.07	2.00	2.90 ± 0.15	94.53	8.05
Serum	1.49	0.75	2.23 ± 0.24	99.53	7.70
	1.49	1.50	2.94 ± 0.08	98.46	2.63
	1.49	3.00	4.59 ± 0.64	102.24	7.85
Liver	9.32	5.00	13.64 ± 0.95	95.26	6.18
	9.32	10.00	18.79 ± 1.91	97.27	9.53
	9.32	20.00	28.88 ± 2.75	98.51	7.75

Abbreviations: standard deviation (SD).

**Table 3:** Cu (II) content of pig samples from local markets (*n* = 3).

Tissue	Sample	Detection Level	Sample	Detection Level	Sample	Detection Level
Pork	CQ1	60.96 ± 5.70	CQ11	39.54 ± 2.57	CQ21	66.55 ± 6.42
(μg/kg)	CQ2	51.18 ± 4.06	CQ12	43.72 ± 4.24	CQ22	52.04 ± 3.55
	CQ3	58.34 ± 5.65	CQ13	39.77 ± 3.97	CQ23	49.76 ± 4.39
	CQ4	42.47 ± 2.81	CQ14	42.56 ± 0.98	CQ24	67.96 ± 6.35
	CQ5	38.07 ± 3.14	CQ15	37.31 ± 3.10	CQ25	85.36 ± 8.42
	CQ6	39.35 ± 2.50	CQ16	39.85 ± 2.53	CQ26	54.32 ± 4.75
	CQ7	42.88 ± 4.12	CQ17	51.02 ± 4.53	CQ27	55.91 ± 5.39
	CQ8	38.35 ± 2.82	CQ18	44.38 ± 4.34	CQ28	50.41 ± 4.98
	CQ9	68.12 ± 5.21	CQ19	39.95 ± 3.65	CQ29	66.31 ± 6.13
	CQ10	37.60 ± 3.05	CQ20	65.66 ± 6.42	CQ30	38.88 ± 2.70
Liver	CQ31	1.30 ± 0.04	CQ33	1.04 ± 0.05	CQ34	1.43 ± 0.06
(mg/kg)	CQ32	1.90 ± 0.12	CQ34	1.57 ± 0.17	CQ35	1.90 ± 0.08

## Supplementary Materials

The following are available online at https://www.mdpi.com/article/10.3390/bios11070235/s1, Table S1: Copper concentration in various samples of pig from two copper source feeding groups at three dosage (Mean ± SD, *n* = 3).
