# Supplementary material for: Development of an Immunoassay for the Detection of Copper Residues in Pork Tissues
Source: Biosensors (Basel). 2021 Jul 13;11(7):235. doi: 10.3390/bios11070235 (PMC8301988; doi:10.3390/bios11070235)
Supplement: Supplementary file 1 [file biosensors-11-00235-s001.zip › biosensors-1253587-supplementary.pdf]

Supporting Information

The ICP-MS conditions of Cu detection

The pig samples were prepared for analysis of Cu concentration including wet tissue weighing 0.20 g liver, pork and serum samples into digestion tubes, respectively. Then add appropriate 3 mL nitric acid at 120℃, digest for 40 minutes, then add 1.2 mL hydrogen peroxide for continuing digest of 40 minutes. After the digestion and transfer samples to beakers and dilute with water to a final volume of 30 mL.

Recovery test

The preprocess followed the pretreatment method in 2.3, and the analysis method is shown in 2.4.

Table S1. Copper concentration in various samples of pig from two copper source feeding groups at three dosage (Mean±SD, n=3).

	**Tribasic Copper Chloride**						**Copper Sulfate**					
	Sample number	Con.	Sample number	Con.	Sample number	Con.	Sample number	Con.	Sample number	Con.	Sample number	Con.
Copper level in fodder (mg/kg)		60		110		210		60		110		210
Serum (mg/kg)	S1	1.53±0.05	S7	2.04±0.23	S13	1.90±0.29	S19	0.98±0.15	S25	1.27±0.17	S31	1.36±0.06
	S2	1.45±0.19	S8	2.14±0.20	S14	2.38±0.19	S20	0.92±0.10	S26	1.33±0.14	S32	1.69±0.23
	S3	2.03±0.12	S9	2.12±0.19	S15	1.76±0.15	S21	1.30±0.11	S27	1.32±0.24	S33	1.25±0.12
	S4	1.72±0.27	S10	1.40±0.18	S16	2.03±0.11	S22	1.10±0.14	S28	0.87±0.17	S34	1.42±0.29
	S5	1.90±0.16	S11	2.27±0.33	S17	1.62±0.20	S23	1.20±0.20	S29	1.40±0.11	S35	1.15±0.14
	S6	1.42±0.21	S12	1.64±0.14	S18	2.18±0.43	S24	0.90±0.10	S30	1.02±0.10	S36	1.56±0.08
Muscle (mg/kg)	M1	0.59±0.09	M7	0.48±0.06	M13	1.03±0.13	M19	0.75±0.14	M25	0.56±0.06	M31	0.81±0.09
	M2	0.64±0.06	M8	0.53±0.04	M14	0.94±0.05	M20	0.81±0.07	M26	0.63±0.08	M32	0.74±0.08
	M3	0.39±0.04	M9	0.83±0.08	M15	0.92±0.09	M21	0.49±0.04	M27	0.99±0.12	M33	0.72±0.68
	M4	0.53±0.06	M10	0.66±0.08	M16	1.23±0.12	M22	0.67±0.08	M28	0.79±0.03	M34	0.97±0.12
	M5	0.56±0.09	M11	0.33±0.04	M17	0.54±0.07	M23	0.71±0.10	M29	0.39±0.07	M35	0.42±0.04
	M6	0.43±0.05	M12	0.57±0.08	M18	0.88±0.12	M24	0.55±0.07	M30	0.68±0.08	M36	0.69±0.05
Liver (mg/kg)	L1	3.43±0.52	L7	6.20±0.38	L13	19.95±2.02	L19	5.07±0.44	L25	7.95±0.79	L31	33.46±3.63
	L2	4.41±0.33	L8	5.58±0.39	L14	21.76±1.27	L20	6.13±0.75	L26	9.59±0.78	L32	30.33±2.03
	L3	5.35±0.63	L9	8.26±0.58	L15	13.25±0.99	L21	4.10±0.56	L27	7.37±1.22	L33	40.55±4.93
	L4	3.75±0.24	L10	5.08±0.93	L16	16.8±2.52	L22	5.58±0.40	L28	11.77±0.65	L34	35.15±3.04
	L5	4.53±0.45	L11	6.17±0.35	L17	13.63±1.43	L23	6.45±0.72	L29	6.50±0.74	L35	29.26±1.80
	L6	4.76±0.22	L12	5.05±0.62	L18	15.88±1.80	L24	8.10±0.28	L30	10.09±1.03	L36	42.14±3.36

Con.: concentration.
